# A Turn-On Fluorescence-Based Fibre Optic Sensor for the Detection of Mercury

**DOI:** 10.3390/s19092142

**Published:** 2019-05-09

**Authors:** T. Hien Nguyen, Tong Sun, Kenneth T. V. Grattan

**Affiliations:** Photonics and Instrumentation Research Centre, City University of London, London EC1V 0HB, UK; t.sun@city.ac.uk (T.S.); K.T.V.Grattan@city.ac.uk (K.T.V.G.)

**Keywords:** optical fibre sensor, fluorescent sensor, mercury sensor, ion imprinted polymer, coumarin dye

## Abstract

The design, development, and evaluation of an optical fibre sensor for the detection of Hg^2+^ in aqueous media are discussed in detail in this paper. A novel fluorescent polymeric material for Hg^2+^ detection, based on a coumarin derivative (acting as the fluorophore) and an azathia crown ether moiety (acting as the mercury ion receptor), has been synthesized. The fluorophore was covalently immobilized onto the fibre surface by polymerisation using the ion imprinting technique and exhibited a significant increase in fluorescence intensity in response to Hg^2+^ via a photoinduced electron transfer (PET) mechanism. The sensor provided a response over a concentration range of 0–28 µM with an acceptable response rate of around 11 min and a recovery rate of around 30 min in a Tris-EDTA buffer solution. A detection limit of 0.15 µM was obtained with a possibility of improvement by changing the thickness of the polymer layer and using a more sensitive detector. High-quality performance is seen through a high selectivity for Hg^2+^ over other metal ions, excellent photo-stability and reversibility which was also demonstrated, making this type of sensor potentially well suited for in-situ monitoring of mercury in the environment.

## 1. Introduction

Mercury pollution in soil due to mining and industrial activities poses a serious problem across the world both from an economic and health perspective [1,2,3]. Common methods for the remediation of mercury-contaminated soil include excavation and disposal, but these methods are often costly and crude. Mercury, both in the inorganic form as Hg^2+^ or in the organic form as methyl mercury, once introduced into the body, can accumulate and cause serious irreversible damage to the immune system, the central nervous system, and kidneys [2,4]. It is also believed that mercury causes various neurodegenerative diseases such as Alzheimer’s disease and Parkinson’s disease [3]. Therefore, the detection of mercury is very important for the protection of human health and the minimization of its exposure in the environment. Such sensors would also provide a warning of exposure and thus act as a trigger for treatment.

In recent years, a number of chemical sensors for the detection of Hg^2+^ have been reported, based either on electrochemical methods [5,6,7] or fluorescence [8,9,10,11,12,13,14,15,16,17,18,19,20,21]. However, there are weaknesses with them and their applications, where most are not suitable for use in the field. Biosensors based on whole bacterial cells or bacterial heavy metal binding proteins [22,23], which can be considered as alternative devices, also suffer from certain limitations due to the fragile and unstable nature of the biological recognition elements. Commercial heavy metal sensors for use in soil are very limited and are typically either very expensive or require the extraction of soil prior to its manipulation and analysis, which could allow sample degradation to occur prior to measurement. Consequently, there is a strong industrial need for the development of a low-cost and portable alternative for mercury detection, thus providing a fast screening solution to yield new information on what is an important aspect of improving the environment.

**Figure 1:**
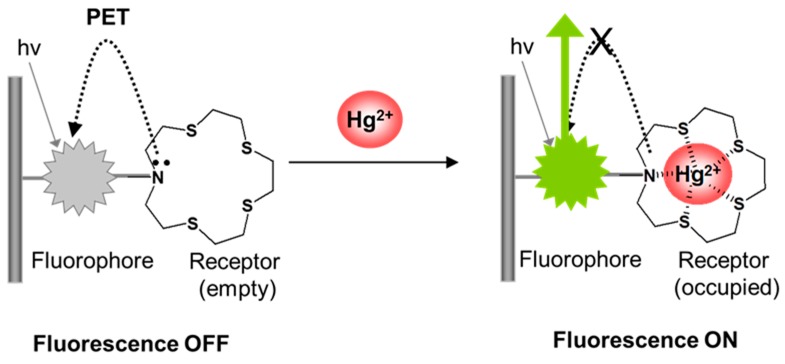
Illustration of fluorescence switching on by Hg^2+^ binding via a photoinduced electron transfer (PET) mechanism.

This work has focused on developing a stable, compact, and portable fibre optic sensing system which is capable of real-time detection of the mercury ion (II). The fibre optic approach used here is familiar for the advantages it offers over conventional means, in terms of small size, immunity to electromagnetic interference, remote sensing capability, resistance to chemicals, and biocompatibility [24]. A novel fluorescent polymeric material for Hg^2+^ detection based on a derivative of coumarin (acting as the fluorophore) and an azathia crown ether moiety (acting as the mercury ion receptor) has been synthesized using the ion imprinting technique and covalently attached to the distal end surface of an optical fibre. Azathia crown ethers have been reported to have a strong ability to coordinate with heavy and transition metal ions and have previously been used as receptors in the design of fluorescent sensors for Hg^2+^ [9,25]. However, the signalling mechanism employed in those sensors was based on intramolecular charge transfer (ICT), leading to the quenching of fluorescence upon analyte binding. It is strongly desirable that the fluorescence modulation is in the ‘off-on’ direction, since this will lead to the best signal-to-noise characteristics and avoid potential false positive responses due to degradation of the system. In this sensor design, the presence of the amine significantly reduces the fluorescence of the fluorophore due to the quenching of its fluorescence by the nitrogen lone pair electrons through a photoinduced electron transfer (PET). Upon complex formation with Hg^2+^, the nitrogen lone pair electrons are donated to Hg^2+^, which therefore abolishes or tremendously reduces the fluorescence quenching. Consequently, binding of Hg^2+^ switches on fluorescence, as illustrated in Figure 1.

## 2. Materials and Methods

### 2.1. General

All chemicals were of analytical grade, purchased from Sigma-Aldrich and were used without further purification. All solvents used were of HPLC grade from Fisher Scientific. All aqueous solutions were prepared using distilled deionized water. ^1^H and ^13^C NMR spectra were recorded on a Bruker Avance Neo 700 or a Bruker Avance 500 spectrometer. Mass spectra were run by the positive ion electrospray (ES) mode on an Agilent LC connected to an Agilent 6510 QTOF mass spectrometer or electron ionisation (EI) mode on a Thermo Finnigan MAT900xp mass spectrometer. IR spectra were recorded on a Bruker Alpha Fourier transform infrared spectrophotometer and were run neat. Melting points were recorded using a Stuart SMP30 melting point apparatus and were uncorrected. Absorption and fluorescence measurements of aqueous solutions containing fluorophores were carried out on a PerkinElmer Lambda 35 spectrophotometer and a Horiba Jobin Yvon Fluoromax-4 spectrofluorometer system with FluorEssence^TM^ as the driving software, respectively. Refractive indices were measured on an Abbe refractometer. Quantum yields of fluorescence were determined using quinine sulfate as the standard (Φ = 0.55) [26,27].

### 2.2. Synthesis

**4-Chloromethyl-7-bromocoumarin (1):** H_2_SO_4_ (80%, 40 mL) was pre-cooled in an ice bath. 3-bromophenol (1.73 g, 10 mmol) was added, followed by ethyl 4-chloroacetoacetate (2.3 g, 1906 µL, 14 mmol) in portions. The mixture was stirred at r.t under Ar for 22 h, after which it was poured into ice water (50 mL) and left stirred for a further 30 min. The white precipitate formed was filtered, washed with H_2_O, dried over phosphorus pentoxide, and recrystallised from EtOH to afford **1** (1.87 g, 68%) as a white solid, mp 211 °C; IR (neat) *ν*_max_ (cm^−1^) 3065, 1736 (C=O), 1598, 1395, 1271, 1243, 1173, 1151, 1081; ^1^H-NMR (500 MHz, CDCl_3_) δ(ppm): 7.56 (d, 1H, H8, *J*_8,6_ = 1.84 Hz), 7.54 (d, 1H, H5, *J*_5,6_ = 8.51 Hz), 7.47 (dd, 1H, H6, *J*_6,5_ = 8.37 Hz, *J*_6,8_ = 1.68), 6.58 (s, 1H, H3), 4.64 (s, 2H, -CH_2_Cl); ^13^C-NMR (DMSO) δ(ppm): 159.1 (C2), 153.8 (C4), 150.2 (C9), 127.5 (C6), 126.9 (C8), 125.1 (C7), 119.8 (C5), 116.4 (C10), 115.8 (C3), 41.1 (-CH_2_-); MS (EI): Calcd. *m*/*z* = 271.89501 (C_10_H_6_O_2_BrCl). Found *m*/*z* = 271.89465 (M^+^). 

**1,4,7,10-tetrathia-13-azacyclopentadecane (2): 2** was prepared similarly to the method reported in literature [9] as white crystals, mp 66 °C; ^1^H-NMR (500 MHz, CDCl_3_) δ(ppm): ppm 2.89 (t, 4H, NC***H***_2_, *J =* 6.16 Hz), 2.87–2.78 (m, 12H), 2.75 (t, 4H, NCH_2_C***H***_2_, *J =* 6.16 Hz), 1.67 (s, 1H, NH); ^13^C-NMR (CDCl_3_) δ(ppm): 48.8, 33.1, 32.9, 32.8, 32.5; MS (ES^+^): Calcd. *m*/*z* = 284.0635 (C_10_H_22_NS_4_). Found *m*/*z* = 284.0640 (M + H^+^).

**7-bromo-4-((1,4,7,10-tetrathia-13-azacyclopentadecan-13-yl)methyl)-coumarin (3):** A mixture of **1** (150 mg, 0.55 mmol), **2** (142 mg, 0.50 mmol), potassium carbonate (200 mg, 1.45 mmol), and posstasium iodide (25 mg, 0.15 mmol) in dry MeCN (10 mL) was heated under argon at 80 °C for 18 h. Triethylamine (0.25 mL) was then added and the mixture was heated at 85 °C for a further two hours. After cooling to room temperature, H_2_O was added to dissolve the inorganic salts. The mixture was filtered to remove insoluble materials. EtOAc was then added. The organic phase was washed with H_2_O (2 × 25 mL) and saturated aqueous NaCl (25 mL), dried over MgSO_4_, filtered, and concentrated in vacuo. The resulting orange residue was chromatographed on silica gel using CH_2_Cl_2_-EtOAc (7:3, *v*/*v*) as an eluent to give **3** (180 mg, 69%) as a pale yellow solid, mp 120 °C; IR (neat) *ν*_max_ (cm^−1^) 3051, 2848, 2821, 1720 (C=O), 1622, 1594, 1445, 1423, 1309, 1237, 1194, 1105; ^1^H-NMR (700 MHz, CDCl_3_) δ(ppm): 7.69 (d, 1H, H5, *J*_5,6_ = 8.53 Hz), 7.52 (d, 1H, H8, *J*_8,6_ = 1.84 Hz), 7.40 (dd, 1H, H6, *J*_6,5_ = 8.51 Hz, *J*_6,8_ = 1.86 Hz), 6.63 (s, 1H, H3), 3.79 (s, 2H, C4C***H*_2_**-), 2.84 (dd, 4H, NC***H***_2,_ *J =* 8.74, 6.33 Hz), 2.83‒2.75 (m, 12H), 2.70 (dd, 4H, NCH_2_C***H***_2,_ *J =* 8.71, 6.32 Hz); (DEPT) ^13^C-NMR (CDCl_3_) δ(ppm): 127.65 (C6), 125.76 (C5), 120.51 (C8), 115.39 (C3), 56.39 (C4***C***H_2_-), 54.76 (N***C***H_2_), 32.25, 32.86, 32.76, 30.40 (NCH_2_***C***H_2_); MS (ES^+^): Calcd. *m*/*z* = 520.0103 (C_20_H_27_O_2_NS_4_Br). Found *m*/*z* = 520.0104 (M + H^+^).

**7-vinylphenyl-4-((1,4,7,10-tetrathia-13-azacyclopentadecan-13-yl)methyl)-coumarin(4): 4** was prepared from **3** via a Suzuki coupling reaction with vinylphenylboronic acid. A mixture of **3** (170 mg, 0.32 mmol), 4-vinylphenylboronic acid (71 mg, 0.48 mmol, 1.5 mol equiv), potassium carbonate (164 mg, 1.18 mmol, 3.7 mol equiv), and dioxane (8 mL) was stirred in a two-necked flask at room temperature under argon for 0.5 h. Tetrakis(triphenylphosphine)palladium(0) (18 mg, 0.016 mmol, 5 mol %) was added. A condenser was fitted and the flask was evacuated and filled with argon three times before being heated to 85 °C. The reaction was left at 85 °C in the dark for 18 h. After cooling to room temperature, EtOAc was added. The organic phase was washed with water (3 × 25 mL), saturated aqueous NaCl (25 mL), dried over MgSO_4_, filtered, and concentrated in vacuo to give the crude product, which was purified by flash chromatography on silica gel with CH_2_Cl_2_-EtOAc (8:2, *v*/*v*), as an eluent to give **4** as a yellow solid. This was further purified by recrystallization from EtOH-EtOAc (5:1, *v*/*v*) to give a yellow solid (90 mg, 52%), mp 155 °C; IR (neat) *ν*_max_ (cm^−1^) 3072, 2909, 2821, 1722 (C=O), 1610, 1422, 1392, 1272, 1157, 1115; ^1^H-NMR (700 MHz, CDCl_3_) δ(ppm): 7.84 (d, 1H, H5, *J*_5,6_ = 8.53 Hz), 7.61 (d, 2H, aromaticH *J* = 8.29 Hz), 7.57 (d, 1H, H8, *J*_8,6_ = 1.75 Hz), 7.53 (d, 2H, aromaticH *J* = 8.25 Hz), 7.53 (dd, 1H, H6, *J*_6,5_ = 8.52 Hz, *J*_6,8_ = 1.75 Hz), 6.77 (dd, 1H, C***H***=CH_2_, *J* = 10.89 Hz, *J* = 17.59 Hz), 6.63 (s, 1H, H3), 5.83 (d, 1H, CH=C***H_a_***H_b_, *J* = 17.93 Hz), 5.33 (d, 1H, CH=CH_a_***H_b_***, *J* = 11.16 Hz), 3.85 (s, 2H, C4C***H*_2_**-), 2.88 (dd, 4H, NC***H***_2,_ *J =* 8.82, 6.34 Hz), 2.84–2.77 (m, 12H), 2.73 (dd, 4H, NCH_2_C***H***_2,_ *J =* 8.80, 6.32 Hz); (DEPT) ^13^C-NMR (CDCl_3_) δ(ppm): 136.23 (***C***H=CH_2_), 127.44 (aromaticC_._), 127.07 (aromaticC_._), 125.02 (C5), 122.86 (C6), 115.12 (C8), 114.96 (CH=***C***H_2_), 114.80 (C3), 56.32 (C4***C***H_2_-), 54.94 (N***C***H_2_), 33.36, 32.86, 32.77, 30.46 (NCH_2_***C***H_2_); MS (ES^+^): Calcd. *m*/*z* = 544.1467 (C_28_H_34_O_2_NS_4_). Found *m*/*z* = 544.1469 (M + H^+^).

### 2.3. Fluorescence Measurements of Free Fluorophore in Solution

Emission spectra were recorded using a 0.8 mL (0.2 × 1 × 4 cm) quartz cuvette. Slit widths were adjusted to give emission below the saturating limit (1,000,000 cps) of the detector, and then the same setting was used for a complete set of experiments. 2.0 mM fluorophore solution was prepared by dissolving 10 × 10^−3^ mmol of the compound in 5.0 mL of MeCN. 2.0 mM metal ion solutions were prepared by dissolving 10 × 10^-3^ mmol of nitrate of chloride salt of the corresponding metal in 5.0 mL of deionized distilled water. Various amounts of Hg^2+^ stock solution and 50 μL of fluorophore solution were added to small glass vials and the samples were made up to a total volume of 2.0 mL with MeCN and deionized distilled water so that the volume ratio of MeCN to H_2_O in the final solution was equal to 3:7. The contents of the vials were transferred into a 0.8 mL cuvette and fluorescence spectra were acquired. 

For the construction of the Job plot [28], stock solutions of the fluorophore and Hg^2+^ were mixed in varying proportions. MeCN and deionized distilled water were then added so that the total concentration of the fluorophore and Hg^2+^ was equal to 50 μM and the volume ratio of MeCN to H_2_O in the final solution was equal to 3:7, with spectra being recorded for each sample.

### 2.4. Sensor Probe Fabrication

The fabrication of the mercury sensing probe requires a multi-step process which is described below. The distal end of a 1000 μm diameter UV multimode fibre (purchased from Thorlabs) was polished in succession with 5 μm, 3 μm, and 1 μm polishing pads (Thorlabs) and washed with acetone to create a clean, polished surface. The distal end was then immersed in a 30:70 (*v*/*v*) mixture of H_2_O_2_ (30%) and H_2_SO_4_ (conc.) (Piranha Solution) for 60 min, rinsed in distilled water for 15 min and dried in an oven at 100 °C for 30 min. This procedure leaves the surface with exposed hydroxyl groups which facilitate bonding of a silane agent. The fibre surface was then modified by silanizing for 2 h in a 10% solution of 3-(trimethoxysilyl)propyl methacrylate in toluene. The fibre was then washed repeatedly with toluene in an ultrasonic bath. Subsequently, it was cured in an oven at 70 °C for 2 h. This procedure functionalizes the fibre surface with polymerizable acrylate groups.

**Figure 2:**
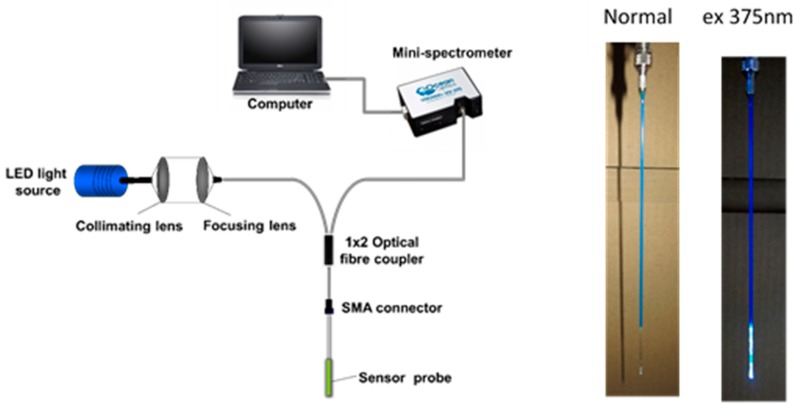
(**Left**): experimental set-up used in the evaluation of the performance of the probe designed. (**Right**): Hg^2+^ probe prepared in this work under normal conditions and when 375 nm UV light was launched to the end of the fibre.

The pre-polymerization mixture was prepared by mixing a solution containing **4** (1.74 mg, 0.0032 mmol) and poly(ethylene glycol) diacrylate (Mn = 575) (30 mg) in *N,N*-dimethylacetamide (500 µL) with a 40% acrylamide solution in water (500 µL) and 2 × 10^−3^ M HgCl_2_ solution in water (160 µL). An ammonium persulfate initiator (10 mg) was then added. The solution was purged thoroughly with argon for 2 min. A small volume of the solution was placed into a capillary tube via a syringe and the distal end of the fibre was inserted. They were sealed quickly with PTFE tape and polymerized in an oven at 75 °C for 15 min. This procedure forms a polymer layer of the dye which is covalently bound to both the cylindrical surface and the distal end surface of the fibre. However, only the polymer on the distal end surface is responsible for the fluorescence signal which is produced by direct excitation from the light source. The polymer on the side plays no role in the sensing process since evanescent wave excitation is eliminated by keeping the cladding of the fibre intact. A typical probe prepared by this procedure is shown in Figure 2 where it can be seen that the distal end of the probe shows a decoloration due to the presence of the fluorophore. The sensor tip was placed in a Tris-EDTA buffer solution (10 mM Tris-HCl; 1 mM EDTA) overnight, followed by repeated washing with distilled water to remove the template (Hg^2+^), and all unreacted materials as well as the excess amount of polymer formed, which was not directly bound to the fibre. The probe was then stored in a cool and dark place until use. 

### 2.5. Sensor Probe Fabrication

The set-up used for the measurements undertaken to calibrate the probe is as presented in Figure 2, where light from a 3 mW LED (Roithner Lasertechnik), emitting at a center wavelength of 375 nm is coupled through a multimode UV/Visible fibre (with hard polymer cladding, 1000 µm silica core and numerical aperture NA of 0.37, Thorlabs), using collimation and focusing lenses (Comar), into one branch of a 2 × 1 multimode fibre coupler (Ocean Optics). The other end of the fibre coupler is connected, through a SMA connector, to the sensor probe with the active sensing region being located at the distal end of the fibre. Following interaction of Hg^2+^ with the active region, a portion of the total light emitted from the sensing layer is collected and guided through the other end of the fibre coupler to an Ocean Optics USB2000 spectrometer, the output from which is then displayed on a computer screen. 

## 3. Results and Discussion

### 3.1. Design and Synthesis of the Mercury Sensitive Fluorophore

The fluorescent molecule used in this work was designed, using photoinduced electron transfer (PET) as the mechanism to translate a cation binding event into a fluorescence signal. This followed the basic design principle of a chromophore–spacer–receptor architecture where the receptor in its empty state is capable of quenching the fluorescence of the attached fluorophore by PET, but in its occupied state is not. The receptor (quencher) unit is separated electronically from the fluorophore. A coumarin chromophore was chosen for this application as coumarins are widely used as laser dyes for single-molecule fluorescence, and so they are ‘tried and tested’ in terms of the key property of being photo-stable [29,30]. An azathia crown ether was employed as the receptor as this group of ethers has been reported to have a strong ability to coordinate with heavy and transition metal ions, and has previously been used as a receptor in the design of fluorescent sensors for Hg^2+^ [9,25].

**Scheme 1:**
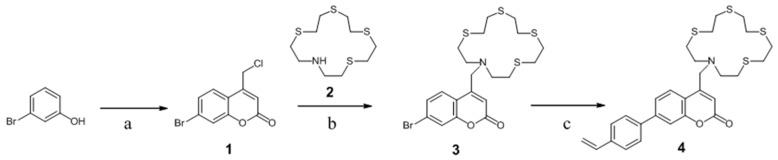
Preparation of mercury sensitive fluorophore styryl (1,4,7,10-tetrathia-13-azacyclopentadecanyl)methyl coumarin (STAMC). (**a**) Ethyl 4-chloroacetoacetate, H_2_SO_4_, H_2_O, r.t, 22 h, 68%; (**b**) vinylbenzylchloride, K_2_CO_3_, KI, MeCN, 80 °C, 18 h, 69%; (**c**) CH_2_=CHC_6_H_4_B(OH)_2_, Pd(PPh_3_)_4_, K_2_CO_3_, dioxane, 85 °C, 18 h, 52%.

The fluorophore was also designed to contains suitable functional group(s) for covalent immobilization of the molecule to a substrate/optical fibre to avoid dye leaching, a common problem resulting from poor immobilization that consequently causes a drifting of the calibration of the probe, which leads to the gradual breakdown of its useful sensing ability [31]. To meet the desired requirements discussed above, a novel polymerisable coumarin, styryl (1,4,7,10-tetrathia-13-azacyclopentadecanyl)methyl coumarin (STAMC, **4**), was prepared in multiple steps starting from a commercially available phenolic compound as outlined in Scheme 1. The Pechmann reaction of a phenol with a β-carbonyl ester is a versatile approach for the synthesis of 4-substituted coumarins. The substitution of chlorine for an azathia crown ether moiety was achieved by performing a reaction with the azathia crown ether **2**. A polymerisable group was introduced into the coumarin structure via a Suzuki coupling of the Br-substituted coumarin **3** with vinylphenylboronic acid using K_2_CO_3_ in dioxane as a base/solvent mixture.

### 3.2. Spectral Properties and Fluorescence Response of the Fluorophore to Metal Ions in Solution

**Figure 3:**
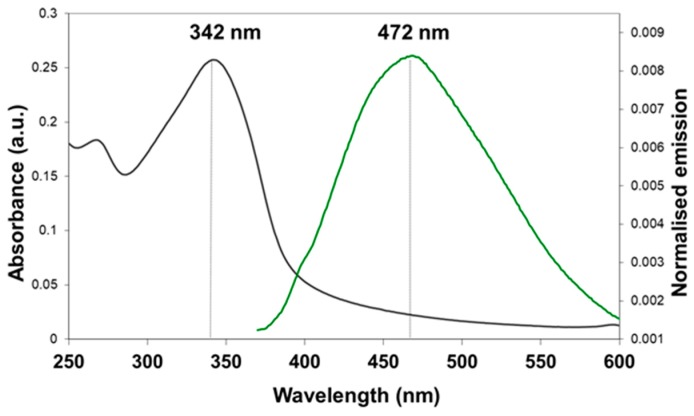
Absorption (**left**) and emission (**right**) spectra of STAMC (10 μM) in H_2_O/MeCN (7:3, *v*/*v*). Emission spectra recorded with λ_ex_ = 342 nm.

Absorption and fluorescence studies of free STAMC (**4**) were performed in H_2_O/MeCN (7:3, *v*/*v*) as the compound is not soluble in H_2_O alone. The absorption spectrum of STAMC shows only one main absorption band in the UV region, at 342 nm (Figure 3). Emission spectrum for the compound recorded in the same solvent using excitation at the absorbance maximum includes one band at 472 nm. It is noted that STAMC exhibits a very large Stokes shift (the difference in wavelength between the absorption and the fluorescence spectral peaks) of 130 nm, which is very important for the sensor system design to minimize the interference of the excitation light with the fluorescence emission. The quantum yield of STAMC was calculated to be 1.5%, which is relatively low as the fluorescence of the compound is quenched by the nitrogen lone pair through PET. 

**Figure 4:**
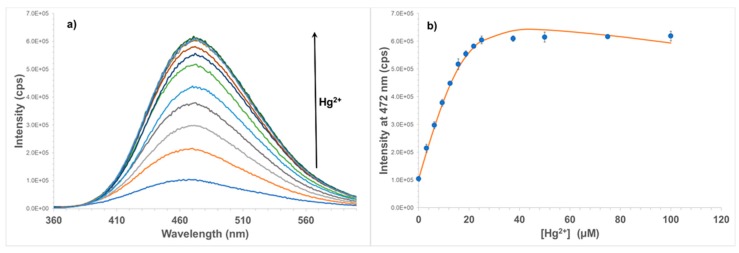
(**a**) Emission spectra of STAMC (50 µM, λ_ex_ = 345 nm) in H_2_O/MeCN (7:3, *v*/*v*) with the addition of Hg^2+^ (0 to 100 µM). (**b**) Titration plot at 472 nm. The red solid line shows the computer fit.

Adding Hg^2+^ to STAMC in the solution resulted in a significant increase in fluorescence intensity of the fluorophore. Figure 4 shows the changes in fluorescence of STAMC with different concentrations of Hg^2+^. It can be seen that the emission intensity at 472 nm increases sharply, nearly 6-fold after the addition of only 25 µM (0.5 equivalent) of Hg^2+^. No significant change was observed after further addition of Hg^2+^, indicating that an approximate saturation was reached. This suggests that the interactions of the fluorophore with Hg^2+^ are very strong, but not on a 1:1 basis, rather on a 2:1 basis.

**Figure 5:**
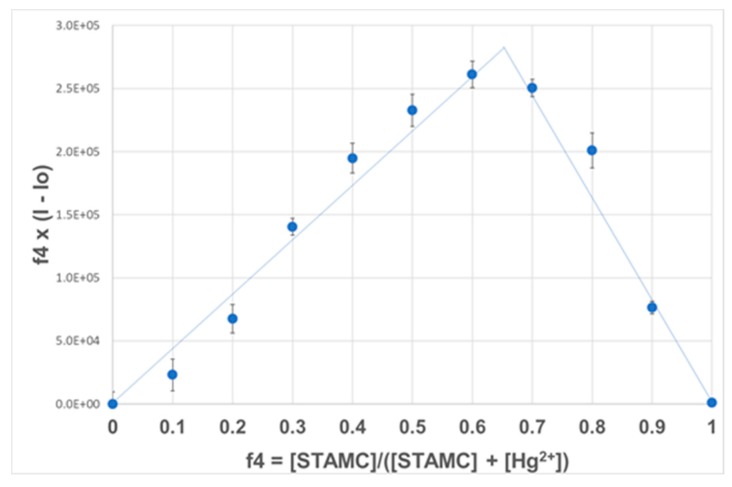
Job plot for the association between STAMC and Hg^2+^ in H_2_O/MeCN (7:3, *v*/*v*).

The binding stoichiometry of STAMC and Hg^2+^ was determined using Job’s method of continuous variation [28]. The total concentration of STAMC and Hg^2+^ was maintained constant at 50 µM, with a continuous variation of the molar fraction of STAMC. Figure 5 shows the fluorescence intensity variation at 472 nm as a function of f4 (f4 being the fraction of STAMC, **4** in a fixed total concentration ([STAMC] + [Hg^2+^] = 50 µM). The sharp peak in the plot at f4 = 0.67 confirms that the dominant species is the 2:1 complex, which is also in agreement with the titration data. 

The titration data were then fitted to the 2:1 association model using the BindFit program [32], giving an extremely high binding constant K_2:1_ of 1.29 ± 0.13 × 10^9^_._ There are a number of complicating factors, including self-association and self-quenching of the fluorophore, which means the true association constant is likely to be less than this. Nonetheless, this indicates that the association between STAMC and Hg^2+^ is very strong and STAMC is an excellent receptor for Hg^2+^. 

**Figure 6:**
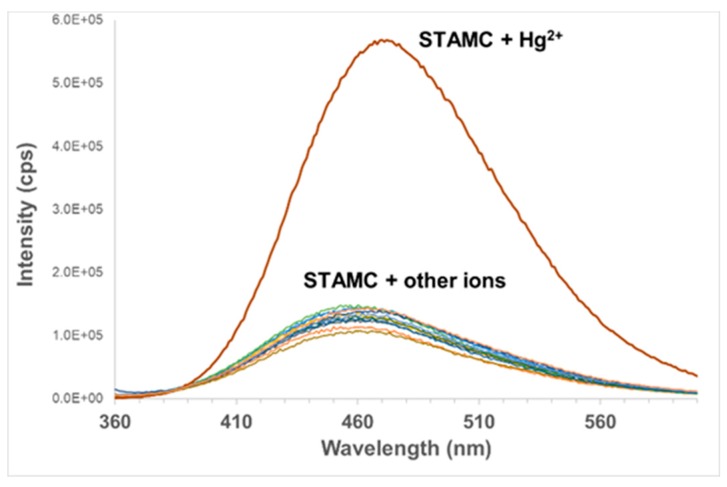
Emission spectra of STAMC (50 µM, λ_ex_ = 345 nm) in H_2_O/MeCN (7:3, *v*/*v*) in the presence of 1 equiv of different metal ions.

The selectivity of STAMC for Hg^2+^ was studied in H_2_O/MeCN (7:3, *v*/*v*). Different heavy metal, transition metal, alkali earth, and alkali ions including Cd^2+^, Pb^2+^, Cu^2+^, Ni^2+^, Zn^2+^, Ca^2+^, Mg^2+^, Co^2+^, Cr^3+^, Mn^2+^, Fe^3+^, Al^3+^, Ag^+^, and Na^+^ were used for the investigation. The concentration of all the metal ions was fixed at 50 µM (1 equiv) where only Hg^2+^ caused a dramatic change in the fluorescence intensity, indicating that STAMC is highly selective for the detection of Hg^2+^ (Figure 6), which is a matter of necessity for an excellent chemosensor. 

### 3.3. Sensor Probe Fabrication

STAMC was covalently immobilized onto the fibre surface by polymerization, in an approach similar to the method previously reported [33]. Several factors need to be taken into consideration when preparing this type of sensing material, designed to work in aqueous/moisture environments: (i) the material has to be sufficiently hydrophilic to allow for water and ions transport, (ii) it should not be too rigid (preventing accessibility of ions to the receptors) nor insufficiently robust, (iii) it has to show long-term stability to allow for multiple measurements. A number of co-monomers (methylmethacrylate, vinylalinine, vinylpiridine, acrylamide), cross-linkers (1,4-bis(acryloyl)piperazine, bisacrylamide, ethylene glycol dimethacrylate, poly(ethylene glycol) diacrylate), solvents (acetonitrile, ethanol, dimethylformamide, *N,N*-dimethylacetamide (DMA), pH 7 phosphate buffer), initiators (ammonium persulfate, azobisisobutyronitrile, diphenyl(2 4 6-trimethylbenzoyl)phosphine oxide), and initiation methods (thermal or photochemical) have been investigated in an attempt to find the most sensitive polymer that satisfies the above requirements. However, all the resulting polymers, despite being very good in quality, showed a very poor sensitivity to Hg^2+^ in comparison to the free fluorophore in solution. This could be because the specific orientation of the metal ion and receptor required for the interactions to happen, particularly when the binding is on a 1:2 basis, is difficult to achieve in polymer when conformational flexibility of the receptor is partly compromised. In addition, self-association and self-quenching of the fluorophore in the polymer also reduces its availability to interact with Hg^2+^ ions. The ion imprinting technique was them employed to create binding sites since the polymer mimics the composition of the pre-polymerisation mixture and maintains the specific orientation of the functional group(s) by the imprinting process. Molecular imprinting has been demonstrated widely as a versatile technique for the preparation of molecular receptors capable of the selective recognition of given target molecules [34,35,36]. However, in this application, ion imprinting was solely used as a method for improving the binding properties and sensitivity of the polymer as the fluorophore is inherently selective. This arrangement enabled the binding sites to match the charge, size, and coordination number of the ion. Moreover, the complex geometry could be preserved through the crosslinking and leaching steps, generating a favourable environment for the template ion rebinding [36]. 

The polymer was prepared directly on the surface of the optical fibre in H_2_O/DMA (1:1, *v*/*v*) using acrylamide as the co-monomer, poly(ethylene glycol) diacrylate (Mn 575) as the cross-linker, and ammonium persulfate as the initiator. The molar amount of fluorescent monomer (STAMC) used was fixed at 1:16 of the cross-linker. More fluorescent monomer would be expected to give more binding sites, but too high a concentration of fluorophore could also result in fluorescence quenching by the inner filter effect. A template:fluorescent monomer ratio of 1:1 or higher was employed to ensure that the fluorescent monomer was fully complexed. Hg^2+^ ions were then removed from the polymer using a Tris-EDTA buffer solution.

### 3.4. Response of the Sensor to Hg^2+^

**Figure 7:**
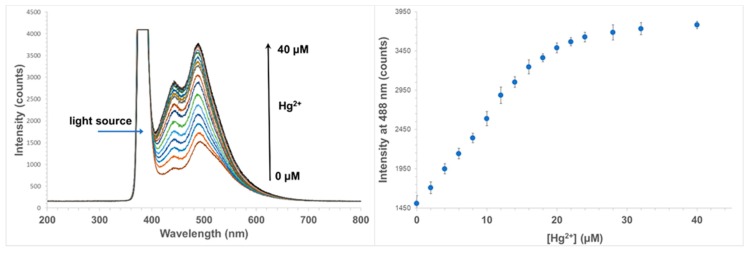
Response of the sensor to Hg^2+^ in the concentration range from 0 to 40 µM in H_2_O (**left**). Titration plot at 488 nm (**right**).

The calibration of the sensor was performed using a series of solutions of mercury chloride in deionized water. The probe was immersed in the Hg^2+^ solutions and the signals were allowed to reach constant values before being recorded. The sensor was rinsed with Tris-EDTA buffer solution (10 mM Tris-HCl; 1 mM EDTA), followed by deionized water between measurements. In a way that is similar to that seen for the free fluorophore, the sensor exhibited an increase in fluorescence intensity with increasing Hg^2+^ concentration in the range of 0–28 µM (Figure 7). At higher concentrations of Hg^2+^, no further change of intensity was observed due to the saturation of all available binding sites. It has also been noted that the emission peak of the immobilized form of the fluorophore is slightly ‘red shifted’ compared to that of its free form in solution. It seems that this probably can be attributed to the change in the polarity of the microenvironment. The lower limit of detection of the system may vary since it depends on the type and sensitivity of the detector used. With the Ocean Optics mini-spectrometer used in this work, the lowest concentration of Hg^2+^ that can cause a distinguishable change in fluorescence intensity is around 0.15 µM. The sensitivity of the sensor is suitable for use in soil where the total concentration of Hg normally ranges from 0.5 to 3000 ppm (2.5 µM to 15 mM) with acceptable limits of up to 12 ppm [37,38,39]. In certain applications where a lower detection range is required, a thinner layer of the sensing material can be fabricated together with the use of a more sensitive detector, which to some extent could lower the detection limit of the sensor. 

### 3.5. Reusability and Photostability 

**Figure 8:**
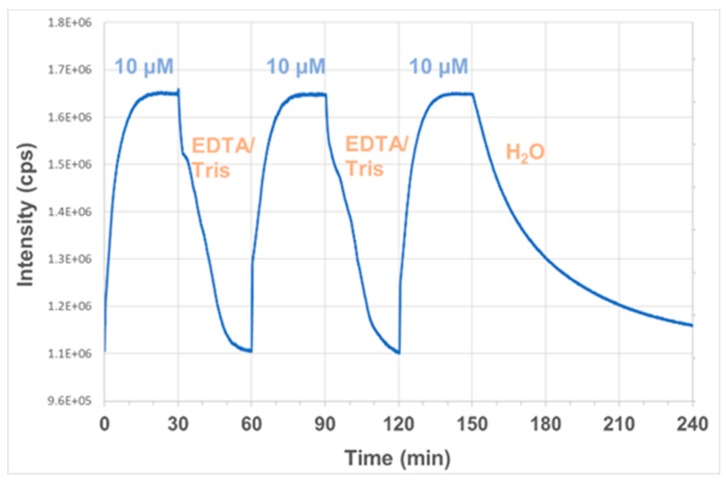
Forward and reverse dynamic response of the sensor probe with the addition of 10 µM Hg^2+^ and removal of Hg^2+^ using a Tris-EDTA buffer solution (10 mM Tris-HCl; 1 mM EDTA) or pure water for three cycles (λ_ex_ = 375 nm, λ_em_ = 440 nm).

Sensor reusability is important for the development of a tool that can allow multiple, rapid and real-time measurements in the field. Consequently, a range of known mercury binders and washing agents were screened to identify a method for probe regeneration. TPEN (*N*,*N*,*N*′,*N*′-tetrakis(2-pyridylmethyl)ethylenediamine) has been reported as a good ion chelator to remove metal ions and restore the systems back to baseline levels [8,40,41]. However, in this study, a Tris-EDTA buffer solution proved superior to TPEN in terms of regeneration time and efficiency so it was used for further investigation. Figure 8 shows the dynamic response of the sensor obtained from the spectrofluorometer to a step change from no Hg^2+^ present (0 µM) to 10 µM Hg^2+^ in H_2_O, and back again by removal of the mercury using a Tris-EDTA buffer solution (10 mM Tris-HCl; 1 mM EDTA) for three cycles. It can be seen from the figure that the metal binding process is completely reversible and the Tris-EDTA solution restores fluorescence of the sensor back to the baseline level. The sensor can also be recovered in H_2_O but the process requires a greater amount of time, almost two hours to attain a 90% recovery compared to just 15 min with the use of EDTA. 

The response time of the sensor upon adding Hg^2+^ can also be seen from Figure 8. Although around 75% of the total signal change occurred within five minutes, it took around 11 min for the sensor to attain 95% of the total change and 18 min to reach equilibrium. The recovery of the sensor after Hg^2+^ binding required a greater amount of time, almost 30 min, to restore the sensor back to the baseline level using the said Tris-EDTA solution. A stronger EDTA buffer solution can be used to make the process faster but too strong solutions may damage the system.

**Figure 9:**
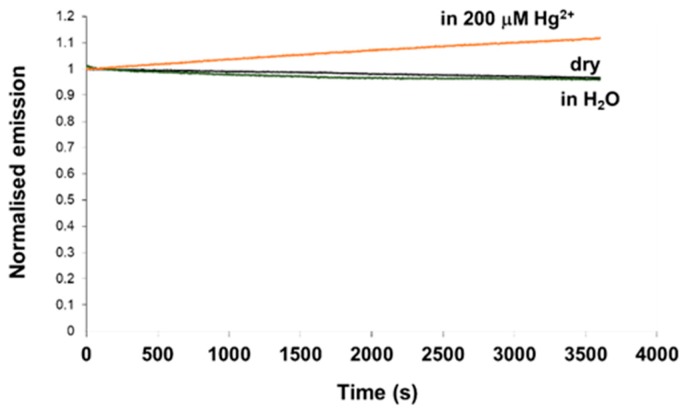
Fluorescence intensity of the sensor probe (in dry state, in H_2_O, and in a 200 µM Hg^2+^ solution) as function of time during 60 min of continuous illumination by light from a high power Xe lamp (λ_ex_ = 375 nm, λ_em_ = 440 nm). Fluorescence intensities are normalised to the initial emission of the probe for each data set.

Photostability is one of the critical properties of fluorescent sensors and thus requires careful investigation. In order to test the photostability of the material, the probe was coupled into the fluorimeter through a dichroic mirror using a fibre bundle. The excitation light (at a wavelength of 375 nm) was launched to the distal end of the probe illuminating the sensing material with light from the intense, high power Xe lamp of the fluorimeter continuously for 1 h. The fluorescence intensity data from the probe were collected over that period and displayed. Figure 9 shows the fluorescence intensities of the sensor probe in dry state, in H_2_O, and in a 200 µM Hg^2+^ solution as function of time during 60 min of continuous illumination by light from the Xe lamp. It was interesting to observe that the intensity of fluorescence was reduced by 3%–4% for the dry state and in H_2_O over the time investigated, and with the high flux of photons onto the probe. However, under the same conditions, a small increase in fluorescence intensity was seen for the similar probe, prepared in exactly the same way using the same polymer, which was immersed in a 200 µM Hg^2+^ solution (after equilibrium being reached). The reason for this is unclear. It could be because the photo decomposed product of Hg^2+^-fluorophore complex is more fluorescent than the original fluorophore. It should be noted that in actual measurements, the material is not excited continuously, but only for 30 s each time when data need to be collected, after the sensor reaches its equilibrium, by a much weaker 3 mW LED light source. Therefore, a little photobleaching of the material when being illuminated by a strong light source should not cause any significant problem for the monitoring. When compared to the results of other materials, this still offers excellent performance: the decrease observed in the fluorescence intensity was 65% for carboxyfluorescein and 10%‒13% for iminocoumarin derivatives, again after 60 min of continuous illumination using a mercury lamp [42]. Thus, an important conclusion is that the material prepared using the coumarin fluorophore and synthesized specifically for this application in this work possesses superior photostability, a feature that is critically important with excitation of sensor probes by high intensity solid state sources.

## 4. Conclusions

In this paper, a novel fibre optic sensor system for the detection of Hg^2+^ in aqueous media has been reported. The sensing mechanism was based on fluorescence turn-on of a coumarin (acting as the fluorophore) bearing an azathia crown ether moiety (acting as the mercury ion receptor) in the presence of Hg^2+^ via photoinduced electron transfer (PET). The fluorophore was covalently immobilized onto the fibre surface by polymerisation using the ion imprinting technique and exhibited a significant increase in fluorescence intensity in response to Hg^2+^ in the µM concentration range. The sensor has also demonstrated a high selectivity for Hg^2+^ over other metal ions. A washing protocol was identified for sensor regeneration, allowing the probe to be re-used. Laboratory tests demonstrated that the sensor possesses excellent photostability, showing very little photo-bleaching after 60 min of continuous illumination using a high power Xe lamp. Therefore, the sensor system developed is potentially suited for in-situ long term monitoring of Hg^2+^. Further optimisation of the sensor design and packaging is ongoing for the next stage field tests and the results will be reported in future work. The approach developed in this work can also be used for the preparation of sensors for other heavy metals by substituting a suitable receptor for the mercury receptor-zathia crown ether.
